# Relacin, a Novel Antibacterial Agent Targeting the Stringent Response

**DOI:** 10.1371/journal.ppat.1002925

**Published:** 2012-09-20

**Authors:** Ezequiel Wexselblatt, Yaara Oppenheimer-Shaanan, Ilana Kaspy, Nir London, Ora Schueler-Furman, Eylon Yavin, Gad Glaser, Joshua Katzhendler, Sigal Ben-Yehuda

**Affiliations:** 1 Institute for Drug Research, School of Pharmacy, The Hebrew University of Jerusalem, Jerusalem, Israel; 2 Department of Microbiology and Molecular Genetics, Institute for Medical Research Israel-Canada (IMRIC), The Hebrew University of Jerusalem, Jerusalem, Israel; 3 Department of Developmental Biology and Cancer Research, Institute for Medical Research Israel-Canada (IMRIC), The Hebrew University of Jerusalem, Jerusalem, Israel; Osaka University, Japan

## Abstract

Finding bacterial cellular targets for developing novel antibiotics has become a major challenge in fighting resistant pathogenic bacteria. We present a novel compound, Relacin, designed to inhibit (p)ppGpp production by the ubiquitous bacterial enzyme RelA that triggers the Stringent Response. Relacin inhibits RelA *in vitro* and reduces (p)ppGpp production *in vivo*. Moreover, Relacin affects entry into stationary phase in Gram positive bacteria, leading to a dramatic reduction in cell viability. When Relacin is added to sporulating *Bacillus subtilis* cells, it strongly perturbs spore formation regardless of the time of addition. Spore formation is also impeded in the pathogenic bacterium *Bacillus anthracis* that causes the acute anthrax disease. Finally, the formation of multicellular biofilms is markedly disrupted by Relacin. Thus, we establish that Relacin, a novel ppGpp analogue, interferes with bacterial long term survival strategies, placing it as an attractive new antibacterial agent.

## Author Summary

The development of new antibacterial agents has become the major demand for fighting against pathogenic bacteria. The identification of new unexplored cellular targets in this combat is essential to prevent a possible return to the pre-antibiotic era. Traditional antibiotics target essential cellular components such as ribosomes and cell wall constituents, making them effective mostly during bacterial growth. However, the ability of bacteria to reside in nature at durable stages sets the need to cope with these alternative survival strategies. In this report, we present a novel antibiotic, termed Relacin, which targets the Stringent Response, a process required for the transition into stationary phase, crucial for bacterial survival. Relacin inhibits the abundant bacterial Rel enzymes that synthesize the signaling molecules required to activate the Stringent Response. We found that Relacin perturbs the switch into stationary phase in Gram positive bacteria and leads to cell death. Further, Relacin inhibits sporulation and biofilm formation, additional bacterial long term survival strategies. The ubiquity of Rel enzymes among bacteria, combined with the absence of known homologues in mammalian cells, strengthen the potential of Relacin to turn into a therapeutic antibiotic.

## Introduction

The emergence of multi drug resistant bacteria dictates the need to develop novel antibiotics that target key components of essential bacterial processes. The pleiotropic response to starvation, known as the Stringent Response, provides a potential target, as it is crucial for activation of survival strategies such as stationary phase, sporulation and biofilm formation [1]–[4]. Further, the Stringent Response has been recently shown to mediate antibiotic tolerance in nutrient-limited bacteria [5]. The Stringent Response is induced by the accumulation of the bacterial signaling molecules 5′-triphosphate-3′-diphosphate and 5′-3′-bis-diphosphate, collectively called (p)ppGpp [6]. Synthesis of (p)ppGpp has been characterized as a ribosome-dependent pyrophosphate transfer of the β and γ phosphates from an ATP donor to the 3′ hydroxyl group of GTP or GDP [7].

In Gram negative bacteria (p)ppGpp is mostly synthesized by RelA and hydrolyzed by SpoT, while in Gram positive bacteria a bifunctional enzyme, Rel/Spo, both synthesizes and hydrolyses (p)ppGpp [8], [9]. Upon nutrient deprivation, Rel proteins bind to ribosomes blocked by uncharged tRNA and catalyze the synthesis of (p)ppGpp [10]. It has been proposed that Rel proteins hop between stalled ribosomes in order to achieve the (p)ppGpp concentration required to induce the Stringent Response [10]. A recent report, however, proposes that RelA actually synthesizes ppGpp only after it is dissociated from the ribosome [11]. The Rel proteins comprise two major domains: a catalytic domain located in the N-terminus and a regulatory domain in the C-terminus [12]. Crystal structure analysis of the N-terminal domain of Rel/Spo from *Streptococcus equisimilis* (*S. equisimilis*) revealed two conformations with opposing hydrolase and synthetase states [13]. Further, the N-terminal domain was found to harbor two catalytic subdomains: N-terminal which hydrolyses (p)ppGpp and C-terminal that catalyzes its synthesis [12].

When ppGpp accumulates within the bacterial cell it affects transcription and a plethora of physiological activities [14]–[16]. Indeed, the activation of many stress-induced genes is partially or totally dependent on ppGpp [17], [18]. The importance of (p)ppGpp as a regulator of bacterial survival prompted us to develop a series of non-hydrolyzable ppGpp analogues [19] potentially targeting Rel proteins. Here we present Relacin, a potent inhibitor of Rel proteins. We demonstrate that Relacin inhibits RelA and Rel/Spo *in vitro* and impairs growth, sporulation and biofilm formation in Gram positive bacteria.

## Results

### Relacin inhibits (p)ppGpp production by Rel proteins

**Figure 1:**
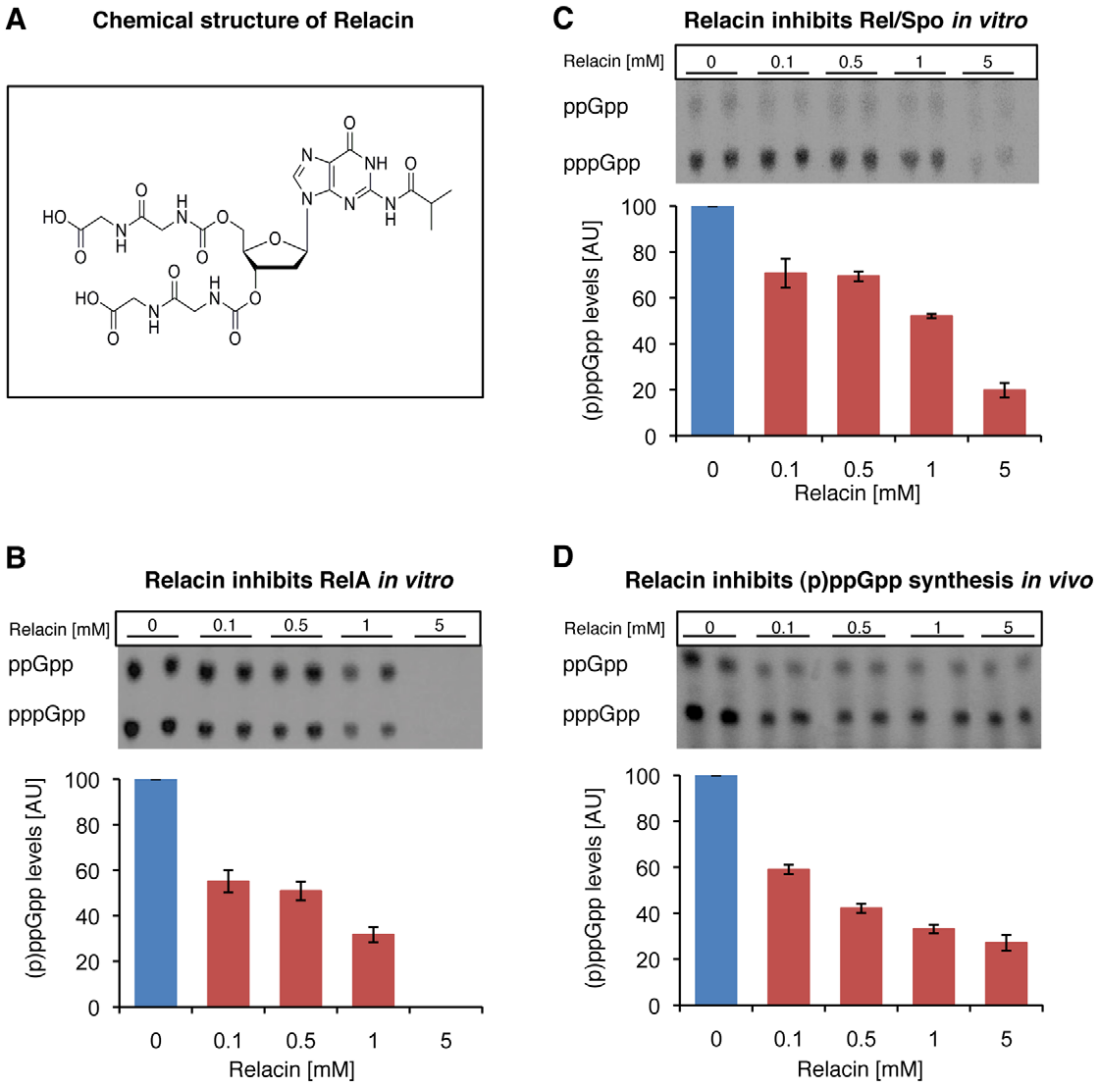
Relacin inhibits the activity of Rel proteins. (**A**) Chemical structure of Relacin. (**B–C**) Relacin inhibits (p)ppGpp synthesis *in vitro*. Representative autoradiograms of PEI thin-layer chromatography showing a decrease in labeled (p)ppGpp synthesized from α-^32^P-GTP precursor by purified RelA (*E. coli*) (B) or Rel/Spo (*D. radiodurans*) (C) with increasing concentrations of Relacin, as indicated (see Materials and Methods). Shown is the average of duplicates of a representative experiment. Error bars represent the range. (**D**) Relacin inhibits (p)ppGpp synthesis in living *B. subtilis* (PY79) cells. The accumulation of (p)ppGpp in response to amino acid starvation, induced by the addition of SHX, was monitored in the absence or presence of increasing concentrations of Relacin, as indicated. The (p)ppGpp level was determined using PEI thin-layer chromatography as in (B–C) (see Materials and Methods). Shown is the average of duplicates of a representative experiment. Error bars represent the range.

Based on the Rel/Spo crystal structure [13], we designed Relacin (Figure 1A), a 2′-deoxyguanosine-based analogue of ppGpp, in which the original pyrophosphate moieties at positions 5′ and 3′ were replaced by glycyl-glycine dipeptides linked to the sugar ring by a carbamate bridge (see Text *S1*; Figure S1A and S1B). Modeling the binding of Relacin to the Rel/Spo synthetase site shows that it occupies a considerable volume of the binding pocket and forms a range of hydrogen bonds and hydrophobic interactions (Figure S1C), providing a structural basis for the inhibitory effect of Relacin.

To investigate the biological activity of Relacin, we first evaluated its inhibitory potential on the (p)ppGpp synthetase activity of RelA and Rel/Spo purified from *Escherichia coli* (*E. coli*) and *Deinococcus radiodurans* (*D. radiodurans*), respectively. Relacin was shown to inhibit both Rel proteins in a dose-dependent manner. Remarkably, at the highest Relacin concentration, the Rel enzymes from Gram negative and positive bacteria were inhibited by approximately 100% and 80%, respectively (Figure 1B and 1C). Notably, the synthesis of ppGpp and pppGpp by both Rel proteins was similarly inhibited (Figure S2A and S2B).

**Figure 2:**
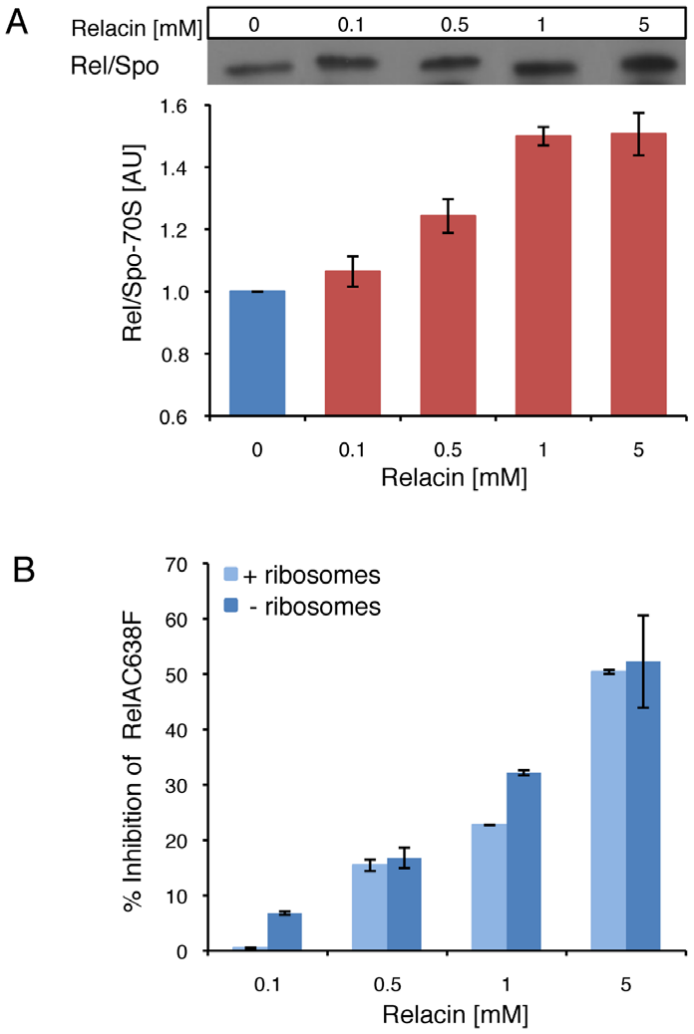
The effect of Relacin on Rel-ribosomes interaction. (**A**) Relacin inhibits dissociation of Rel/Spo from the ribosome. The relative amount of Rel/Spo (*D. radiodurans*) bound to purified ribosomes was quantified following the addition of increasing levels of Relacin. Rel/Spo molecules associated with 70S complexes were detected by Western blot analysis (see Materials and Methods). Histogram indicates the average of two independent biological repeats. Error bars represent the range. (**B**) Ribosome independent inhibition of (p)ppGpp synthesis. The constitutively active, ribosome-independent RelAC638F (*E. coli*) protein was treated with increasing concentrations of Relacin, as indicated (see Materials and Methods) in the presence or absence of isolated ribosomes. Shown is the average of duplicates of a representative experiment. Error bars represent the range.

Next, we examined the effect of Relacin on the interaction between Rel/Spo and stalled ribosomes. Ribosomes purified from *D. radiodurans* were incubated with Rel/Spo in the presence of increasing concentrations of Relacin, and the relative amount of Rel/Spo molecules associated with 70S complexes was examined. Western blot analysis revealed that Relacin increases the levels of Rel/Spo locked on the ribosomes (Figure 2A), suggesting that the presence of Relacin reduces the pool of protein molecules available for (p)ppGpp re-synthesis [10]. To further investigate whether ribosomes are actually required for Relacin activity, we took advantage of a RelA mutant protein (RelAC638F), which exerts its activity in a ribosome-independent manner. Relacin was equally able to inhibit the mutant protein (Figure 2B), indicating a direct Relacin-RelA interaction.

We then examined the influence of Relacin on (p)ppGpp production in living cells upon induction of the Stringent Response. To this end, cells of the Gram positive spore forming bacterium *Bacillus subtilis* (*B. subtilis*) were incubated with Relacin and treated with serine hydroxamate (SHX) to simulate amino acid starvation [20], [21]. Subsequently, the accumulated levels of (p)ppGpp were monitored from cell extracts. In line with the inhibitory activity observed *in vitro*, Relacin markedly reduced (p)ppGpp production *in vivo* (Figure 1D). Interestingly, although Relacin was found to completely inhibit the activity of purified RelA from the Gram negative bacterium *E. coli* (Figure 1B), no obvious effect of the compound on bacterial (p)ppGpp synthesis was observed (Figure S2C). This is most likely due to the inability of Relacin to penetrate the *E. coli* cell and reach its target.

### Relacin reduces survival of Gram positive bacteria

**Figure 3:**
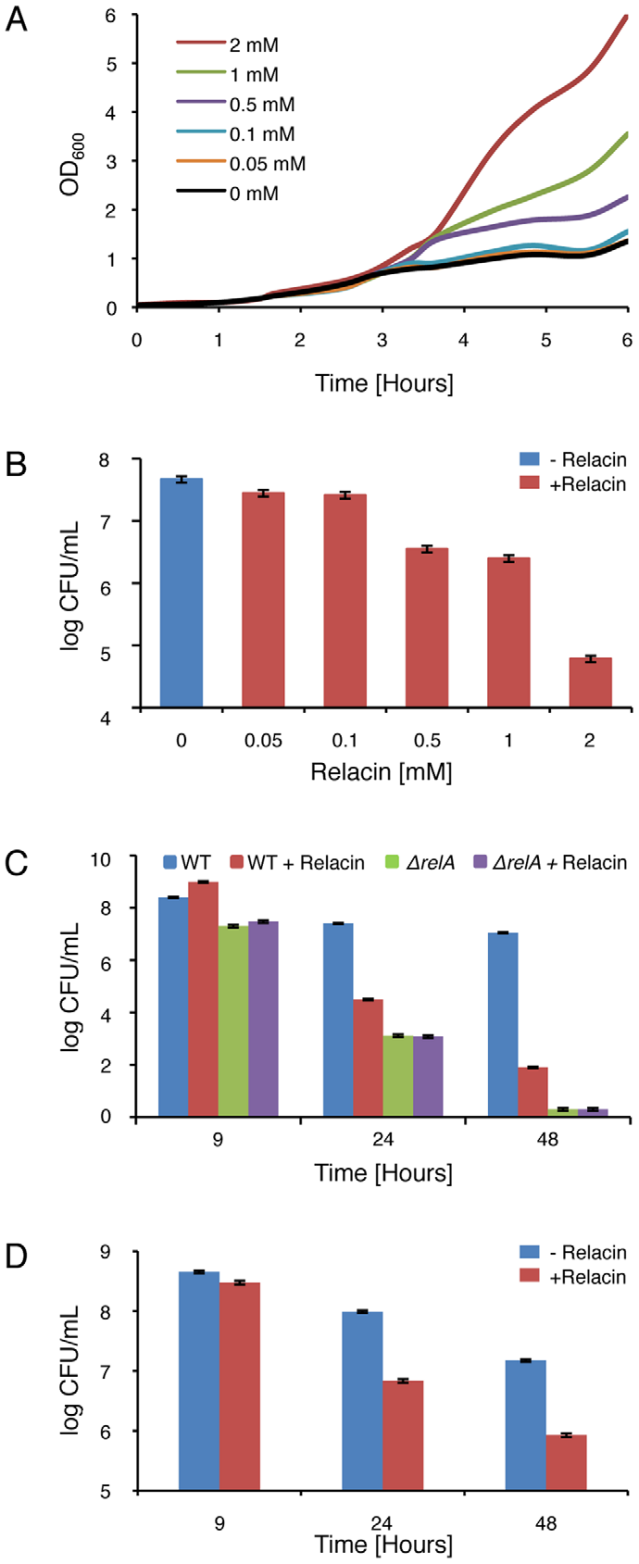
Relacin affects bacterial growth and survival. (**A**) Relacin influences entry into stationary phase. Shown are growth curves of wild type *B. subtilis* (PY79) cells grown in CH medium at 37°C in the absence or presence of increasing concentrations of Relacin added at OD_600_ 0.2. (**B**) Relacin exerts a toxic effect. The viability of *B. subtilis* (PY79) cells was evaluated by counting colony forming units (CFU) after 24 hours of incubation in CH medium at 37°C in the absence or presence of increasing concentrations of Relacin added at OD_600_ 0.2. Shown is a representative experiment, in which SD was calculated from at least three repeats for each concentration. (**C**) Long term effect of Relacin treatment. The effect of Relacin (2 mM) on the viability of wild type *B. subtilis* (PY79) cells or *ΔrelA* (ME215) cells was measured. Cells were incubated in CH medium at 37°C, and viability was determined by counting colony forming units (CFU). Relacin was added at OD_600_ 0.2. Shown is a representative experiment, in which SD was calculated from at least three repeats for each point. (**D**) The toxic effect of Relacin on GAS. The effect of Relacin (2 mM) on the viability of wild type GAS (JRS4) cells, incubated in THY medium at 37°C, was evaluated as in (C).

Having ascertained that Relacin affects the production of (p)ppGpp *in vivo* by *B. subtilis* cells and given the vital role of the Stringent Response in bacterial survival, we investigated the impact of Relacin on cell growth and viability. Interestingly, in the presence of Relacin, cells exhibited an extended logarithmic phase as indicated by the increase in OD_600_ values, implying that they failed to properly enter into stationary phase (Figure 3A). Of note, a similar phenomenon was observed for *spoT* null mutant of *Helicobacter pylori* [22]. This failure led to substantial dose-dependent cell death after 24 hours, with an estimated IC_50_ of 200 µM, as monitored by the reduction in colony forming units (Figures 3B and S3). Moreover, after 48 hours the deleterious effect of Relacin persisted, reducing the number of colonies by approximately five orders of magnitude relative to untreated cultures (Figure 3C). A similar viability pattern was observed in untreated *B. subtilis* cells lacking Rel/Spo (Figure 3C), suggesting that this enzyme is indeed the main target for Relacin action. Consistent with this observation, the survival of the mutant strain was not affected by the addition of Relacin (Figure 3C). Notably, the effect of Relacin on survival is not likely to be dependent on spore formation, as only few spores, if any, were present in untreated cultures. On the other hand, the appearance of dead cells as well as disintegrated cells was largely increased within the treated population over time (Figure S4). Consistent with the inability of Relacin to perturb (p)ppGpp production in *E. coli* (Figure S2C), no effect on growth and viability was detected in these cells.

The biological activity of Relacin was further explored in non-spore-forming Gram positive bacteria. Treating the Group A streptococcus (GAS) with Relacin revealed that, although growth rate was only slightly affected, cell viability was largely reduced after 24 hours (Figure S5A and S5B). This toxic effect was enhanced after 48 hours (Figure 3D) and was associated with the formation of very small colonies. Additionally, as observed for *B. subtilis*, entering stationary phase was perturbed by Relacin in the extremely slow growing bacterium *D. radiodurans* (Figure S5C). Furthermore, the addition of Relacin to *D. radiodurans* cells diminished bacterial viability, as indicated by plating efficiency assay carried out after 56 and 72 hours of incubation (Figure S5D). Thus, we established that Relacin functions as an antibacterial agent that impairs entry into stationary phase and causes bacterial death.

### Relacin perturbs long term survival strategies

**Figure 4:**
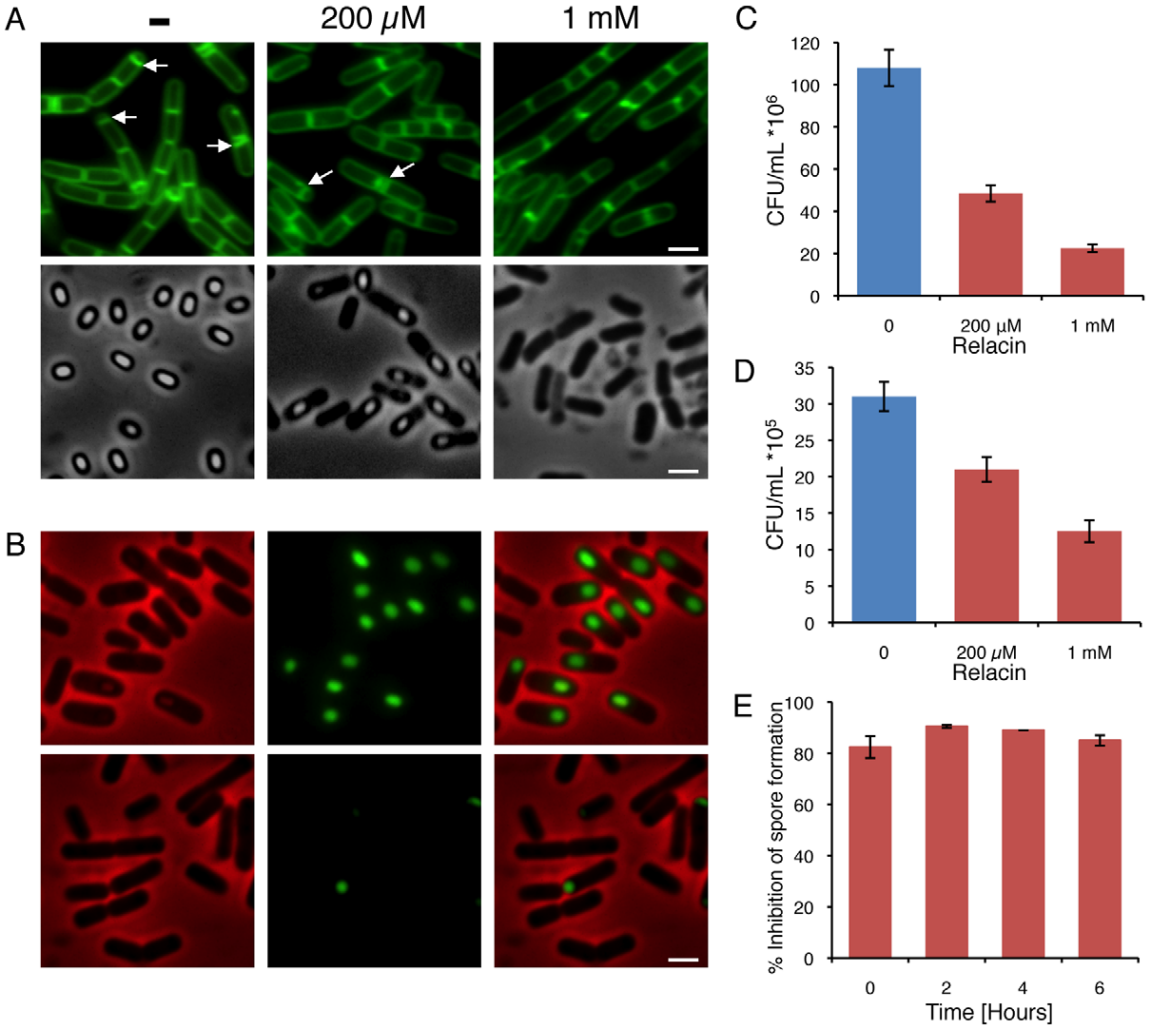
Relacin influences the sporulation process in *Bacilli*. (**A**) Relacin inhibits sporulation. Microscopy images of sporulating wild type *B. subtilis* (PY79) cells in the absence or presence of Relacin, added at time 0 of sporulation at the indicated concentrations. Upper panels: cells at t = 2 hr of sporulation stained with the fluorescent membrane dye FM1–43. Arrows indicate position of polar septa. Lower panels: phase contrast images of cells at t = 24 hr of sporulation. Scale bars correspond to 1 µm. (**B**) Relacin inhibits expression of the mid-sporulation protein SpoIIQ. Fluorescence microscopy images of *B. subtilis* (PE128) cells harboring *spoIIQ-gfp* at t = 4 hr of sporulation, in the absence (upper panels) or presence (lower panels) of Relacin (1 mM), added at time 0 of sporulation. Shown are phase contrast (red), GFP fluorescence (green) and overlay images. Scale bar corresponds to 1 µm. (**C–D**) Relacin inhibits *Bacilli* spore formation. The formation of heat resistant *B. subtilis* (PY79) (C) and *B. anthracis* (Sterne) (D) spores was monitored in the absence or presence of Relacin, added at the indicated concentrations at time 0 of sporulation (see Text *S1*). Shown are representative experiments, in which SD was calculated from at least three repeats for each concentration. (**E**) Relacin added at different time points during sporulation inhibits spore formation. Inhibition of spore formation by wild type *B. subtilis* (PY79) cells was evaluated after addition of Relacin (1 mM) at the indicated time points of sporulation. Inhibition was determined using a heat resistance assay (see Text *S1*) and is expressed relative to untreated cultures. Shown is a representative experiment, in which SD was calculated from at least three repeats for each time point.

In addition to switching into stationary phase some bacteria, such as *Bacilli*, respond to nutrient limitation by producing highly resilient dormant spores as a strategy for long term survival [23]–[25]. Entry into sporulation is triggered by a decrease in the intracellular GTP pools, in part due to conversion of GTP into (p)ppGpp by RelA [26]. At the onset of sporulation, an asymmetric septum is generated, dividing the cell into a nurturing mother cell and a smaller forespore compartment that develops into a spore. Subsequently, the forespore is engulfed by the mother cell to form a fully mature spore. Remarkably, when nutrients become available the spore can rapidly convert into an actively growing cell [23]–[25]. To explore whether Relacin affects sporulation, *B. subtilis* cells were induced to sporulate in the presence or absence of Relacin and sporulation progression was monitored by observing polar septa formation. Indeed, sporulation was largely inhibited, with asymmetric septa exhibited by only 8% and 0.5% of the cells treated with 200 µM and 1 mM of Relacin, respectively. In comparison, 47% of untreated cells displayed polar septa at the same time point (Figure 4A). In line with these observations, Relacin lowered the number of cells expressing early (SpoIIE), middle (SpoIIQ) and late (SspE) sporulation-specific proteins along the process [25] (Figures 4B and S6). Subsequently, a fivefold drop in the formation of mature heat resistant spores was measured at the highest Relacin concentration (Figure 4A and 4C). Remarkably, adding Relacin to sporulating cells at different time points, up to six hours after the induction of sporulation, strongly inhibited spore formation regardless of the time of addition (Figure 4E). These findings indicate that the ppGpp signal is crucial throughout the entire pathway of sporulation, and demonstrate the potency of Relacin to impede this process. Importantly, spore formation in the pathogenic bacterium *Bacillus anthracis*, the causative agent of anthrax disease, was inhibited by Relacin in a similar fashion (Figure 4D), establishing the compound as a general inhibitor of the *Bacilli* sporulation process.

**Figure 5:**
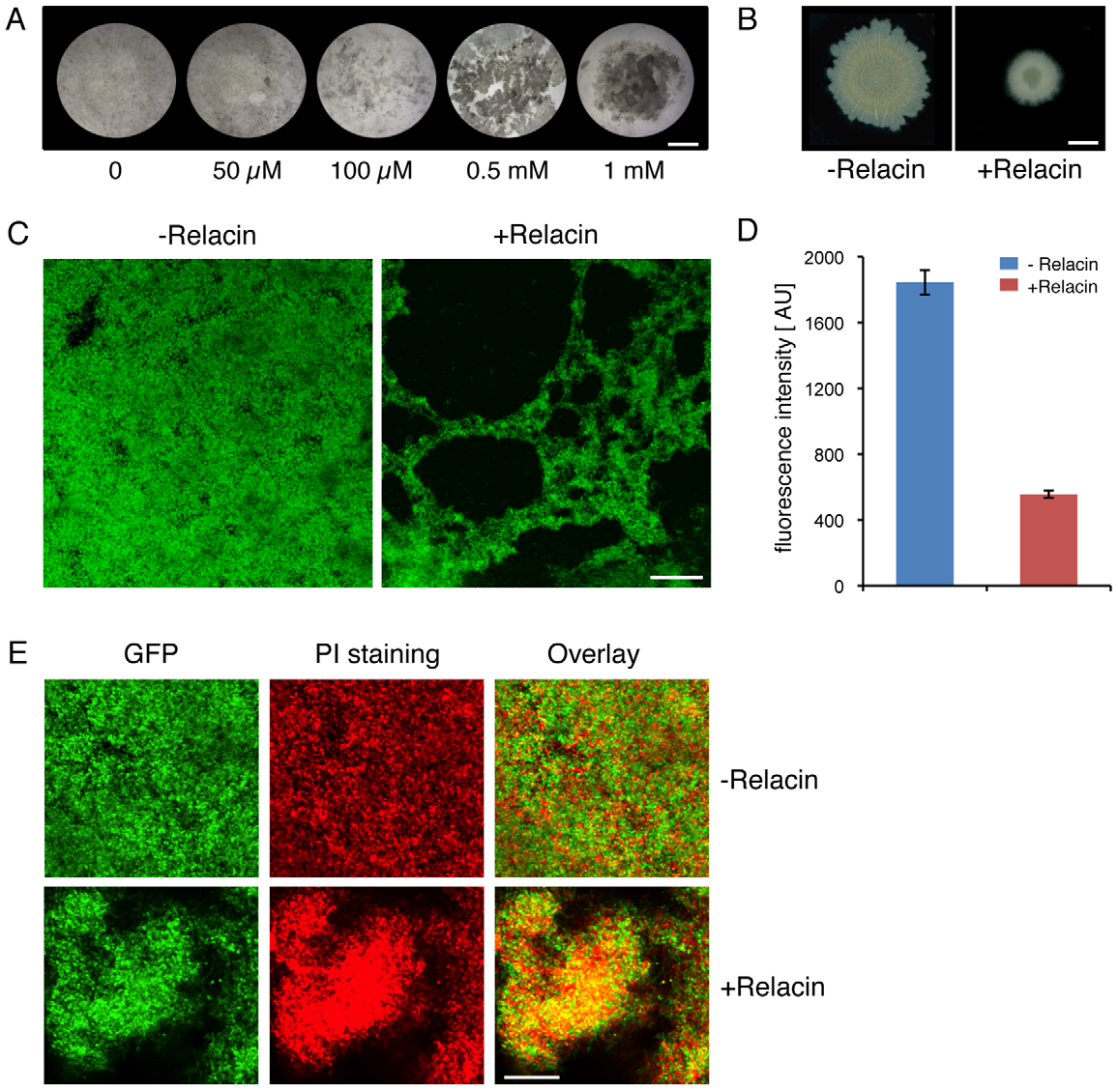
Relacin affects biofilm formation in *Bacillus subtilis.* (**A**) Relacin inhibits pellicle biofilm formation. Wild type *B. subtilis* (3610) cells were induced to form biofilms in liquid standing cultures in the absence or presence of Relacin at the indicated concentrations (see Text *S1*). Cultures were photographed after 3 days. Scale bar corresponds to 5 mm. (**B**) Relacin inhibits biofilm colony formation. Wild type *B. subtilis* (3610) cells were induced to form biofilms on solid medium, in the absence or presence of Relacin (1 mM) (see Text *S1*). Colonies were photographed after 24 hours. Scale bar corresponds to 3 mm. (**C**) Relacin causes biofilm disintegration. *B. subtilis* (YA224) cells harboring P*_rrnE_-gfp* fusion were induced to form biofilm in liquid standing cultures in the absence or presence of Relacin (1 mM) as indicated (see Text *S1*). Biofilms were visualized after 3 days using confocal microscopy (see Materials and Methods). Green signal corresponds to GFP produced from P*_rrnE_*. Scale bar corresponds to 50 µm. (**D**) Relacin reduces biofilm biomass. *B. subtilis* (YA224) cells harboring P*_rrnE_-gfp* fusion were induced to form biofilm in liquid standing cultures in the absence or presence of Relacin (1 mM) as indicated. Biofilm pellicles were disintegrated and cell biomass was evaluated by GFP fluorescence measurements and is displayed in arbitrary units [AU] (see Text *S1*). Shown is the average of two independent biological repeats. Error bars represent the range. (**E**) Relacin leads to cell death within the biofilm. *B. subtilis* (YA224) cells harboring P*_rrnE_-gfp* fusion were induced to form biofilm in liquid standing cultures in the absence or presence of Relacin (1 mM) as indicated (see Text *S1*). After 3 days, biofilms were stained with PI to indicate cell death and observed by confocal microscopy (see Materials and Methods). Shown are GFP fluorescence produced from P*_rrnE_* (green), PI staining (red), and overlay images. Scale bar corresponds to 50 µm.

Since it has been reported that *relA* mutant cells fail to properly form multicellular biofilm structures [2], the effect of Relacin on the ability of *B. subtilis* cells to produce biofilms was evaluated. Indeed, a disrupted pellicle was visualized at the air/liquid interface of standing cell cultures grown in the presence of the compound (Figure 5A). Importantly, the effect on biofilm formation was found to be dose-dependent (Figure 5A). Consistent with this observation, Relacin also inhibited the development of biofilm on solid medium, leading to the formation of colonies with altered morphology that were smaller in size than the untreated ones (Figure 5B). To visualize cell assembly within the biofilm pellicle in higher resolution upon Relacin treatment, we took advantage of a strain harboring the *rrnE* promoter fused to *gfp*. This promoter was found to be constitutively active [27], and therefore reports cell viability and localization. Observing biofilm pellicles by confocal laser scanning microscopy revealed that the untreated cells formed homogeneous biofilm layers, while the treated cell pellicles contained large gaps, indicating their disintegrated state (Figure 5C). Moreover, staining the biofilm with propidium iodide (PI), indicative of unviable cells, showed the signal to be higher within the treated biofilm (Figure 5E). Finally, quantifying GFP fluorescence from recovered pellicles revealed a clear reduction in the viable biomass upon Relacin treatment, as the measured fluorescence level was significantly reduced (Figure 5D). Taken together, we conclude that Relacin interferes with biofilm formation, an alternative bacterial developmental pathway.

## Discussion

In this report, we established Relacin as a novel antibacterial agent. By specifically interfering with the activation of the Stringent Response, Relacin perturbs the switch into stationary phase in several tested Gram positive bacteria and leads to bacterial death. Although Relacin did not affect growth and survival of the Gram negative *E. coli*, it was found to effectively inhibit the *E. coli* RelA *in vitro*, implying that improving the delivery of Relacin to Gram negative bacteria may lead to an effective outcome. Relacin was found to block every tested stage of *B. subtilis* sporulation, proving the essentiality of the Stringent Response throughout this process. Finally, we demonstrate that Relacin affects the production of multicellular biofilm communities, formed in response to challenging conditions. Taken together, we present evidence that Relacin impedes bacterial long term survival pathways, placing the compound as a new promising antibacterial agent.

By utilizing the crystal structure of Rel/Spo from the *S. equisimilis*, we were able to model the interaction of Relacin with amino acid residues located within the Rel/Spo synthetase site. This analysis yielded the identification of a putative binding mode of Relacin, presumably adopting the conformation shown in Figure S1C. In this conformation, Relacin forms a net of hydrogen bonds and hydrophobic interactions that are most likely to provide a more efficient binding in comparison to previously identified inhibitors exhibiting lower activity [19].

Relacin appears to specifically target Rel proteins, as the effect of the compound was nearly undetectable when tested on Rel/Spo mutant cells. Consistently, Relacin activity *in vivo* resulted in a sharp decrease in (p)ppGpp synthesis. Since ppGpp inhibits the enzyme inosine monophosphate dehydrogenase, it causes the cellular GTP levels to decrease [28]. The intracellular levels of GDP/GTP are known to determine the initiation of several developmental pathways such as sporulation and biofilm formation [26], [29], [30] that were indeed shown to be influenced by Relacin. Interestingly, we also observed that Relacin treatment resulted in a large decrease in Rel/Spo ability to dissociate from ribosomes *in vitro*. This deficiency could be explained by the model proposed by Wendrich et al., [10] in which the rapid accumulation of ppGpp during amino acid starvation is attributed to the ability of RelA to ‘hop’ between ribosomes. This potential hopping is probably a consequence of the synthesis of (p)ppGpp that releases RelA from the ribosome, liberating it for another synthesis cycle.

The emergence of bacterial resistance to the current array of antimicrobial agents demands the development of novel strategies to eradicate pathogenic bacteria. The traditional cellular antibiotic targets include ribosomes, cell wall constituents and components of nucleic acids synthesis [31]. These cellular targets are mainly active during the bacterial vegetative phase, making the available antibiotics effective mostly during growth. However, the ability of bacteria to reside in nature within biofilm communities or as durable spores, as well as to become persistent to antibiotic treatment [32], sets the need to tackle these alternative modes. In this regard, Relacin affects specifically the Stringent Response, a pathway crucial for the activation of bacterial survival strategies. Since Relacin can persist for a relatively long period of time, and exert its effect even a few days post addition, it might become a valuable antagonist of these long term survival approaches. Taken together, Relacin may be combined with antibiotics currently in use, to eradicate non-homogenous bacterial populations with cells residing in diverse life cycles.

Cellular components, which are conserved throughout the bacterial kingdom and crucial for cellular survival, provide attractive antimicrobial targets as long as they lack eukaryotic counterparts. One of such targets is the highly conserved bacterial tubulin-like cell division protein FtsZ, which provides the basis for the assembly of the division machinery [33]. Indeed, a promising inhibitor of FtsZ with potent and selective activity against *Staphylococci* has been described [34]. In a similar fashion, the ubiquity of Rel enzymes among bacteria, combined with the absence of known (p)ppGpp synthetases in mammalian cells [35], [36], strengthen the potential of Relacin to turn into a therapeutic antibiotic. The profound influence of Relacin on long term bacterial survival makes it an attractive compound to serve as a scaffold for generating an array of new antibacterial agents.

## Materials and Methods

### Synthesis and modeling of Relacin

Synthesis of Relacin and a structural model for its interaction with Rel/Spo (p)ppGpp synthetase binding pocket are described in details Text *S1*.

### Bacterial growth conditions

Bacterial strains used in this study are described in Table S1. Plasmid construction is described in Text *S1*. All general methods for *B. subtilis* were carried out as described previously [37]. *B. subtilis* cells were grown in hydrolyzed casein (CH) at 37°C [37], unless indicated differently. GAS strain was grown at 37°C without shaking in Todd-Hewitt medium supplemented with 0.2% yeast extract (THY) [38]. *D. radiodurans* R1 cells were grown in TYG which contains: 0.5% tryptone, 0.3% yeast extract and 0.1% glucose at 30°C with shaking. *E. coli* cells were grown at 37°C in LB medium. Cultures were inoculated to an OD_600_ of 0.05 using an overnight culture grown in the same medium, unless indicated differently. Sporulation conditions and biofilm colony and pellicle formation are described in Text *S1*.

### Purification of Rel proteins and crude ribosomes

Purification of RelA or RelA-C638F from *E. coli* (CF9467) harboring *ΔrelA* and over-expressing pQE30-RelA or pQE30-RelA-C638F respectively, was carried out as described previously [19]. Purification of Rel/Spo from *D. Radiodurans* R1 was performed under identical conditions; however, the protein was expressed in *E. coli* BL21 CodonPlus (Stratagene) cells. Of note, Rel/Spo from *D. Radiodurans* R1, is the only known full length active protein purified from Gram positive bacteria. Isolation of crude ribosomes (containing 70S, mRNA, tRNA) from *E. coli* (CF9467) was carried out as described previously [19]. Isolation of crude ribosomes from *D. Radiodurans* was carried out in a similar fashion with the following modifications: *D. radiodurans* R1 cells were grown in LB(+) over night at 30°C, cells were diluted 1∶100 in LB(+) medium and incubated at 30°C for additional 48 hours.

### Measuring (p)ppGpp synthesis *in vitro*

For measuring (p)ppGpp synthesis by RelA, RelA-C638F or Rel/Spo proteins *in vitro*: 1 µg of purified Rel protein, 20 µg of isolated ribosomes and 10 µCi of α-^32^P labeled GTP, were mixed in a buffer [0.5 mM GTP, 4 mM ATP, 50 mM Tris-HCl (pH 7.4), 1 mM DTT, 10 mM MgCl_2_, 10 mM KCl and 27 mM (NH_4_)_2_SO_4_] to a final volume of 20 µL without or with increasing amounts of Relacin as indicated. Reactions were stopped by the addition of 5 µL formic acid. Each reaction was loaded (5 µL) and separated on Cellulose PEI TLC plates (Merck) using 1.5 M KH_2_PO_4_ as mobile phase. Plates were autoradiographed using the Fijix Bas100 PhosphorImager (Japan). (p)ppGpp signal was measured using TINA 2.0 software (Raytest, Strauben-Hardt). The total amount of (p)ppGpp was the sum of signals from ppGpp and pppGpp.

### Measuring (p)ppGpp synthesis *in vivo*

*B. subtilis* (PY79) or *E. coli* (W3110) cells were grown in MOPS glucose minimal medium [39] supplemented with all amino acids except glutamine and glutamate. At OD_600_ 0.1, cells were supplemented with H_3_^32^PO_4_ and incubated for 45 minutes, after which Relacin was added at the indicated concentrations. Cells were incubated for additional 15 minutes. Next, amino acid starvation was induced by adding serine-hydroxamate (SHX, Sigma) 1 mg/mL [20]. Samples were withdrawn ten minutes after addition of SHX and analyzed for their (p)ppGpp content as described above (Measuring (p)ppGpp synthesis *in vitro*).

### Measuring Rel/Spo- 70S association

The reaction was carried out as described above for measuring (p)ppGpp synthesis *in vitro*, without the addition of radiolabeled GTP, with or without increasing amounts of Relacin as indicated. Reactions were centrifuged for 4 hours at 35,000 g (4°C), ribosomal fractions were separated by 12% SDS-polyacrylamide gel electrophoresis, transferred to PVDF membrane (Millipore Bedford) and processed for immunoreaction using mouse anti-His antibody (1∶10,000; Amersham). Immunoreactive proteins were detected using a chemiluminescence kit (Biological Industries) according to the manufacturer's protocol.

### Fluorescence microscopy

Fluorescence microscopy was carried out as previously described [40]. Samples (0.5 mL) of a given culture were removed, centrifuged briefly, and resuspended in 10 µL of PBS×1 (Phosphate-Buffered Saline) supplemented with 1 µg/mL membrane stain FM1–43 or FM4–64 (Molecular Probes, Invitrogen). Cells were visualized and photographed using an Axioplan2 microscope (Zeiss) equipped with CoolSnap HQ camera (Photometrics, Roper Scientific) or an Axioobserver Z1 microscope (Zeiss) equipped with a CoolSnap HQII camera (Photometrics, Roper Scientific). System control and image processing were performed using MetaMorph 7.2r4 software (Molecular Devices).

### Confocal laser scanning microscopy

For observing biofilm pellicles, the medium of 3 day-old pellicles grown in microplates was gently removed and minimal volume of PBS ×1 with or without 10 µg/ml PI (Molecular Probes, Invitrogen) was added. Cells were visualized and photographed using a confocal laser scanning fluorescence microscope LSM700 (Zeiss). System control and image processing were performed using Zen 2009 (Zeiss) and MetaMorph 7.2r4 (Molecular Devices) softwares.

## Supporting Information

Figure S1 **Synthesis of Relacin, a novel ppGpp analogue.** (**A**) Chemical structure of ppGpp. (**B**) Chemical synthesis of Relacin. Reaction conditions: i) Boc anhydride, THF/aqueous sodium bicarbonate, RT, 4 hours 77%; ii) Benzyl alcohol, *p*-toluenesulfonic acid, toluene, reflux, 5 hours, 95%; iii) HOBT/HBTU, DMF, DIEA, RT, overnight. 98%; iv) 50% TFA in DCM, RT, 30 minutes, 86%; v) a) trimethylsilyl chloride, pyridine, 0°C, 1 hour, b) Isobutyric anhydride, RT, 4 hours, 87%; vi) a) CDI, acetonitrile, RT, overnight, b) (**4**), DCM, DIEA, RT, 20 hours, 48%; vii) H_2_, 10% Pd/C, methanol, 3 hours, RT, 30 psi, 80%. (**C**) Structural basis of binding and inhibition of Rel/Spo by Relacin. A putative model describing how Relacin (sticks, colored according to the cpk scheme) binds in the known GDP binding site of Rel/Spo protein from *Streptococcus equisimilis* (shown as transparent white surface and cartoon). Relacin also forms additional contacts with Rel/Spo within the active site. GDP is shown in black lines for comparison, and residues of Rel/Spo that form hydrogen bonding contacts to GDP are shown in stick representation (see Text *S1*). The high affinity of Relacin can be explained by the extensive contacts formed between the ligand and the receptor. The ligand occupies a considerable volume of the binding pocket, including both the GDP binding sites, as well as additional regions. In addition to a range of hydrogen bonds mediated by the overall very polar pocket, the hydrophobic isobutyryl group contacts a defined hydrophobic patch shown as black dots.
(TIF)

Figure S2 **The effect of Relacin on (p)ppGpp synthesis.** (**A**) Relacin inhibits RelA *in vitro*. The relative amount of ppGpp and pppGpp produced by purified RelA (*E. coli*) in the absence or presence of Relacin at the indicated concentrations was calculated from autoradiograms of PEI thin-layer chromatography, corresponding to Figure 1B. Shown is the average of duplicates of a representative experiment. Error bars represent the range. (**B**) Relacin inhibits Rel/Spo *in vitro*. The relative amount of ppGpp and pppGpp produced by purified Rel/Spo (*D. radiodurans*) in the absence or presence of Relacin at the indicated concentrations was calculated from autoradiograms of PEI thin-layer chromatography, corresponding to Figure 1C. Shown is the average of duplicates of a representative experiment. Error bars represent the range. (**C**) Relacin does not inhibit (p)ppGpp synthesis in living *E. coli* cells. The accumulation of (p)ppGpp in response to amino acid starvation, induced by SHX, was monitored in the absence or presence of increasing concentrations of Relacin. The (p)ppGpp level was determined using PEI thin-layer chromatography of radiolabeled (p)ppGpp (see Materials and Methods). Histogram indicates the average of two independent biological repeats. Error bars represent the range.
(TIF)

Figure S3 **Effect of Relacin on survival of** ***B. subtilis*** **grown in minimal medium.** Survival of wild type *B. subtilis* (PY79) cells grown in S7 minimal medium was determined by CFU counting after 9 and 24 hours of incubation in the absence or presence of Relacin (2 mM), as indicated. Relacin was added at OD_600_ 0.2. Shown is a representative experiment, in which SD was calculated from at least three repeats for each point.
(TIF)

Figure S4 **The toxic effect of Relacin is visible.** (**A–B**) *B. subtilis* (PY79) cells were grown in CH medium at 37°C in the absence (upper panels) and presence (lower panels) of Relacin (1 mM), added at OD_600_ 0.2. Cells were stained with viability indicators SYTO9 (green, highlights live cells) and PI (red, highlights dead cells) at 9 hours (A) and 24 hours (B) of incubation. Shown are phase contrast images (left panels) and Live/Dead overlay fluorescence images (right panels). Of note, some of the disintegrated cells were not stained with any of the dyes. Scale bar corresponds to 1 µm.
(TIF)

Figure S5 **Relacin affects growth and survival of Gram positive bacteria.** (**A**) Effect of Relacin on growth of GAS. Shown are growth curves of wild type GAS (JRS4) cells grown at 37°C without shaking in THY medium in the absence or presence of increasing concentrations of Relacin added at OD_600_ 0.2. (**B**) Effect of Relacin on survival of GAS. The effect of Relacin, at the indicated concentrations, on survival of GAS (JRS4) cells grown at 37°C without shaking in THY medium was determined by CFU counting of treated and untreated cultures. Relacin was added at OD_600_ 0.2. Shown is a representative experiment, in which SD was calculated from at least three repeats for each concentration. (**C**) Effect of Relacin on growth of *D. radiodurans*. Shown are growth curves of wild type *D. radiodurans* R1 grown in TYG medium at 30°C in the absence or presence of Relacin at the indicated concentrations added at OD_600_ 0.2. (**D**) Effect of Relacin on *D. radiodurans* survival. The effect of Relacin, at the indicated concentrations, on survival of wild type *D. radiodurans* R1 cells grown at 30°C in TYG medium was determined by CFU counting of treated and untreated cultures. Relacin was added at OD_600_ 0.2. Shown is a representative experiment, in which SD was calculated from at least three repeats for each point.
(TIF)

Figure S6 **Effect of Relacin on the expression of early and late sporulation-specific proteins.** (**A**) Fluorescence microscopy images of *B. subtilis* (SB201) cells harboring *spoIIE-gfp* fusion at t = 2 hr of sporulation, in the absence (upper panels) and presence (lower panels) of Relacin (1 mM), added at time 0 of sporulation. Shown are cells stained with FM4–64 membrane dye (red), SpoIIE-GFP fluorescence (green) and overlay images. Scale bar corresponds to 1 µm. (**B**) Fluorescence microscopy images of *B. subtilis* (ES7) cells harboring *sspE-gfp* fusion at t = 5 hr of sporulation, in the absence (upper panels) and presence (lower panels) of Relacin (1 mM), added at time 0 of sporulation. Shown are phase contrast (red), SspE-GFP fluorescence (green) and overlay images. Scale bar corresponds to 1 µm.
(TIF)

Text S1 **Supplemental information, including one table as well as additional experimental procedures and references.**
(DOCX)
