# Supplementary figures and images for: Relacin, a Novel Antibacterial Agent Targeting the Stringent Response
Source: PLoS Pathog. 2012 Sep 20;8(9):e1002925. doi: 10.1371/journal.ppat.1002925 (PMC3447753; doi:10.1371/journal.ppat.1002925)

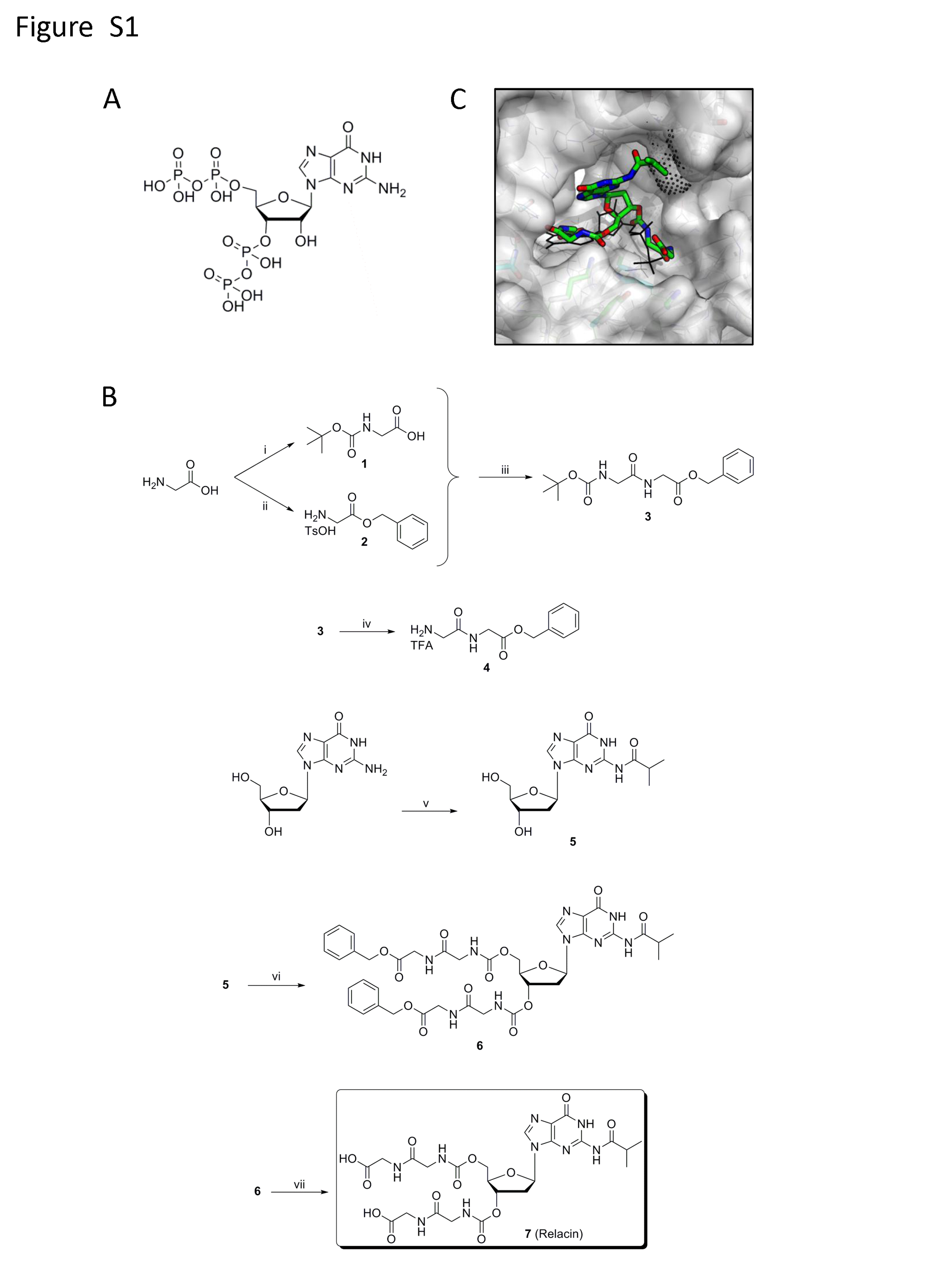

Supplement: Figure S1 — Synthesis of Relacin, a novel ppGpp analogue. (A) Chemical structure of ppGpp. (B) Chemical synthesis of Relacin. Reaction conditions: i) Boc anhydride, THF/aqueous sodium bicarbonate, RT, 4 hours 77%; ii) Benzyl alcohol, p-toluenesulfonic acid, toluene, reflux, 5 hours, 95%; iii) HOBT/HBTU, DMF, DIEA, RT, overnight. 98%; iv) 50% TFA in DCM, RT, 30 minutes, 86%; v) a) trimethylsilyl chloride, pyridine, 0°C, 1 hour, b) Isobutyric anhydride, RT, 4 hours, 87%; vi) a) CDI, acetonitrile, RT, overnight, b) (4), DCM, DIEA, RT, 20 hours, 48%; vii) H2, 10% Pd/C, methanol, 3 hours, RT, 30 psi, 80%. (C) Structural basis of binding and inhibition of Rel/Spo by Relacin. A putative model describing how Relacin (sticks, colored according to the cpk scheme) binds in the known GDP binding site of Rel/Spo protein from Streptococcus equisimilis (shown as transparent white surface and cartoon). Relacin also forms additional contacts with Rel/Spo within the active site. GDP is shown in black lines for comparison, and residues of Rel/Spo that form hydrogen bonding contacts to GDP are shown in stick representation (see Text S1). The high affinity of Relacin can be explained by the extensive contacts formed between the ligand and the receptor. The ligand occupies a considerable volume of the binding pocket, including both the GDP binding sites, as well as additional regions. In addition to a range of hydrogen bonds mediated by the overall very polar pocket, the hydrophobic isobutyryl group contacts a defined hydrophobic patch shown as black dots. (TIF) [file ppat.1002925.s001.tif]

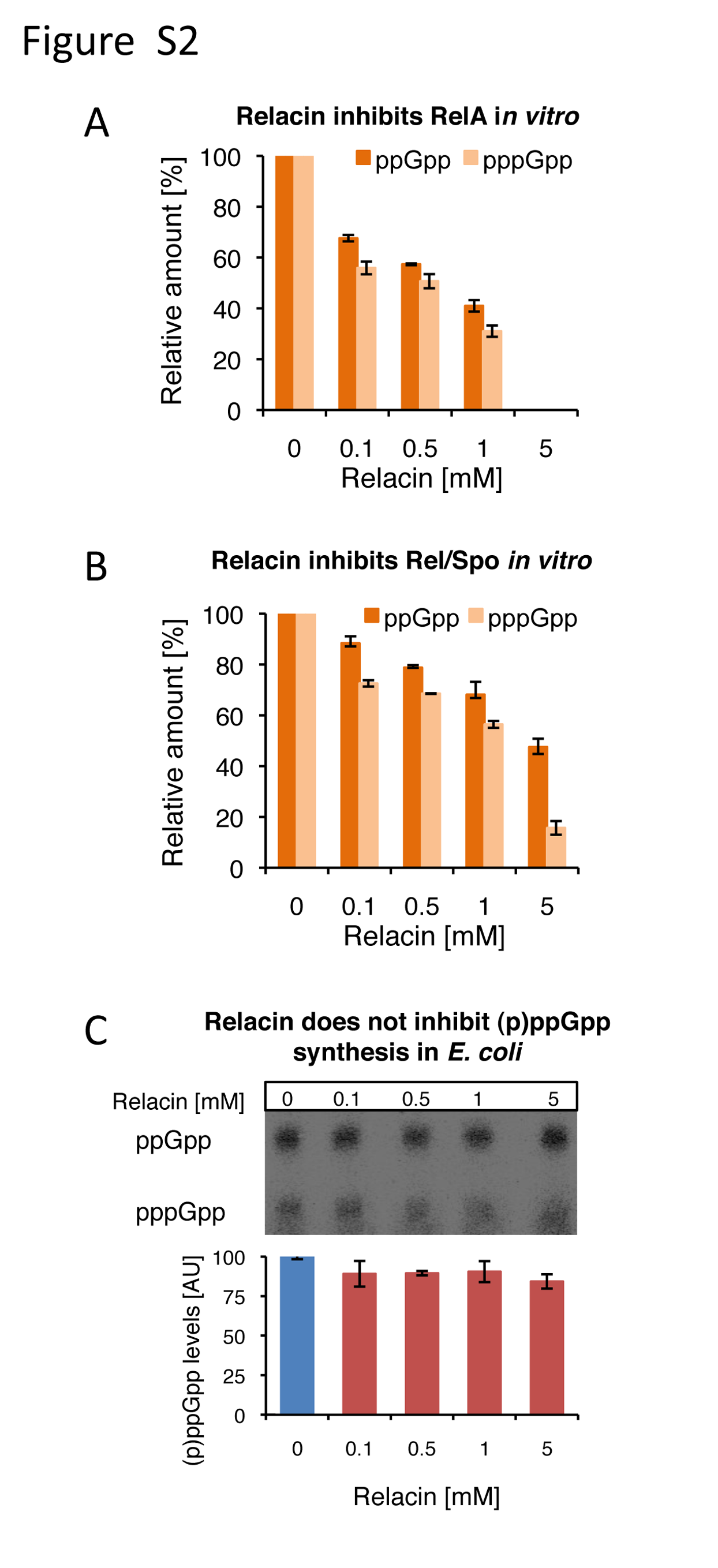

Supplement: Figure S2 — The effect of Relacin on (p)ppGpp synthesis. (A) Relacin inhibits RelA in vitro. The relative amount of ppGpp and pppGpp produced by purified RelA (E. coli) in the absence or presence of Relacin at the indicated concentrations was calculated from autoradiograms of PEI thin-layer chromatography, corresponding to Figure 1B. Shown is the average of duplicates of a representative experiment. Error bars represent the range. (B) Relacin inhibits Rel/Spo in vitro. The relative amount of ppGpp and pppGpp produced by purified Rel/Spo (D. radiodurans) in the absence or presence of Relacin at the indicated concentrations was calculated from autoradiograms of PEI thin-layer chromatography, corresponding to Figure 1C. Shown is the average of duplicates of a representative experiment. Error bars represent the range. (C) Relacin does not inhibit (p)ppGpp synthesis in living E. coli cells. The accumulation of (p)ppGpp in response to amino acid starvation, induced by SHX, was monitored in the absence or presence of increasing concentrations of Relacin. The (p)ppGpp level was determined using PEI thin-layer chromatography of radiolabeled (p)ppGpp (see Materials and Methods). Histogram indicates the average of two independent biological repeats. Error bars represent the range. (TIF) [file ppat.1002925.s002.tif]

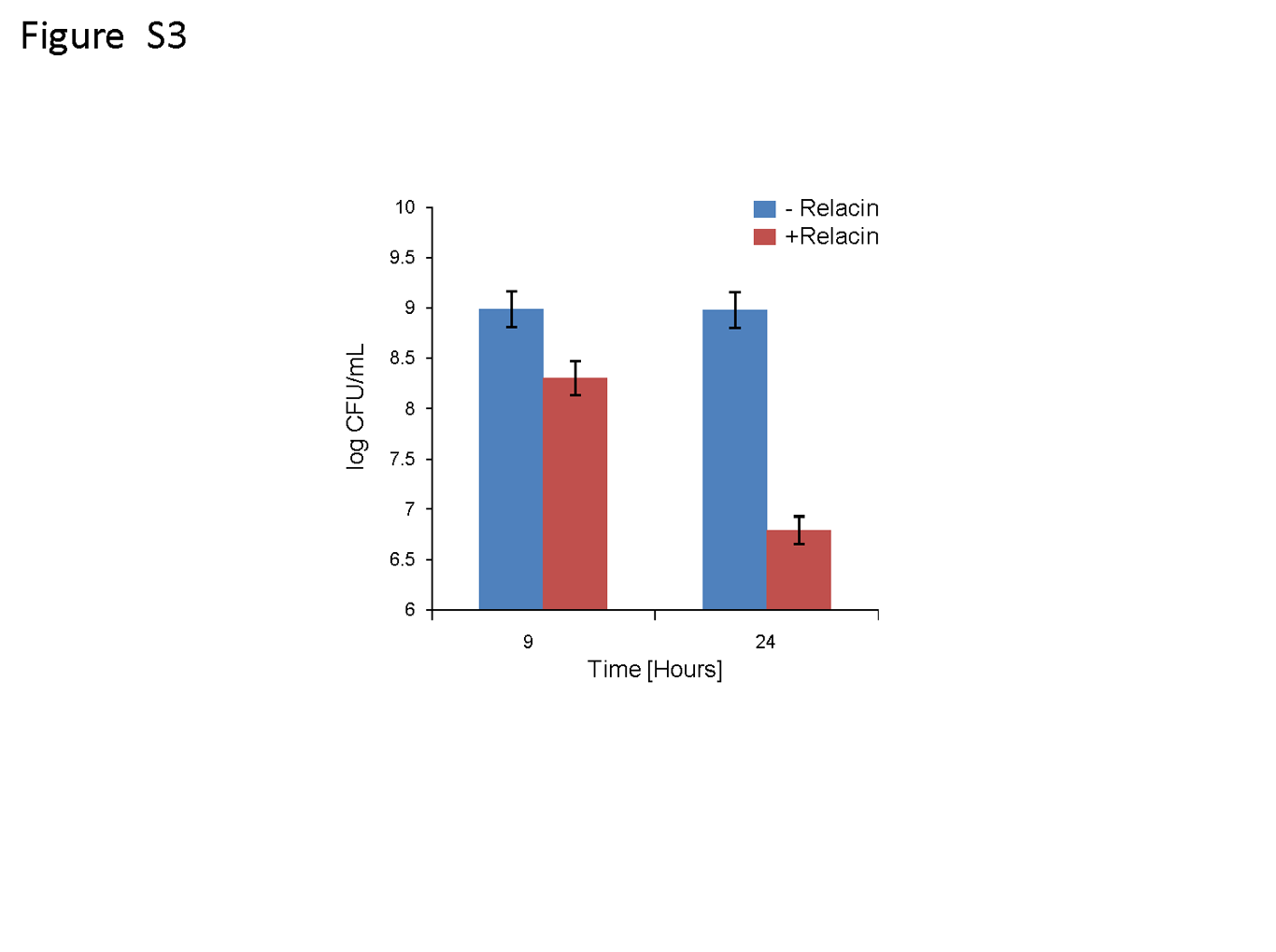

Supplement: Figure S3 — Effect of Relacin on survival of B. subtilis grown in minimal medium. Survival of wild type B. subtilis (PY79) cells grown in S7 minimal medium was determined by CFU counting after 9 and 24 hours of incubation in the absence or presence of Relacin (2 mM), as indicated. Relacin was added at OD600 0.2. Shown is a representative experiment, in which SD was calculated from at least three repeats for each point. (TIF) [file ppat.1002925.s003.tif]

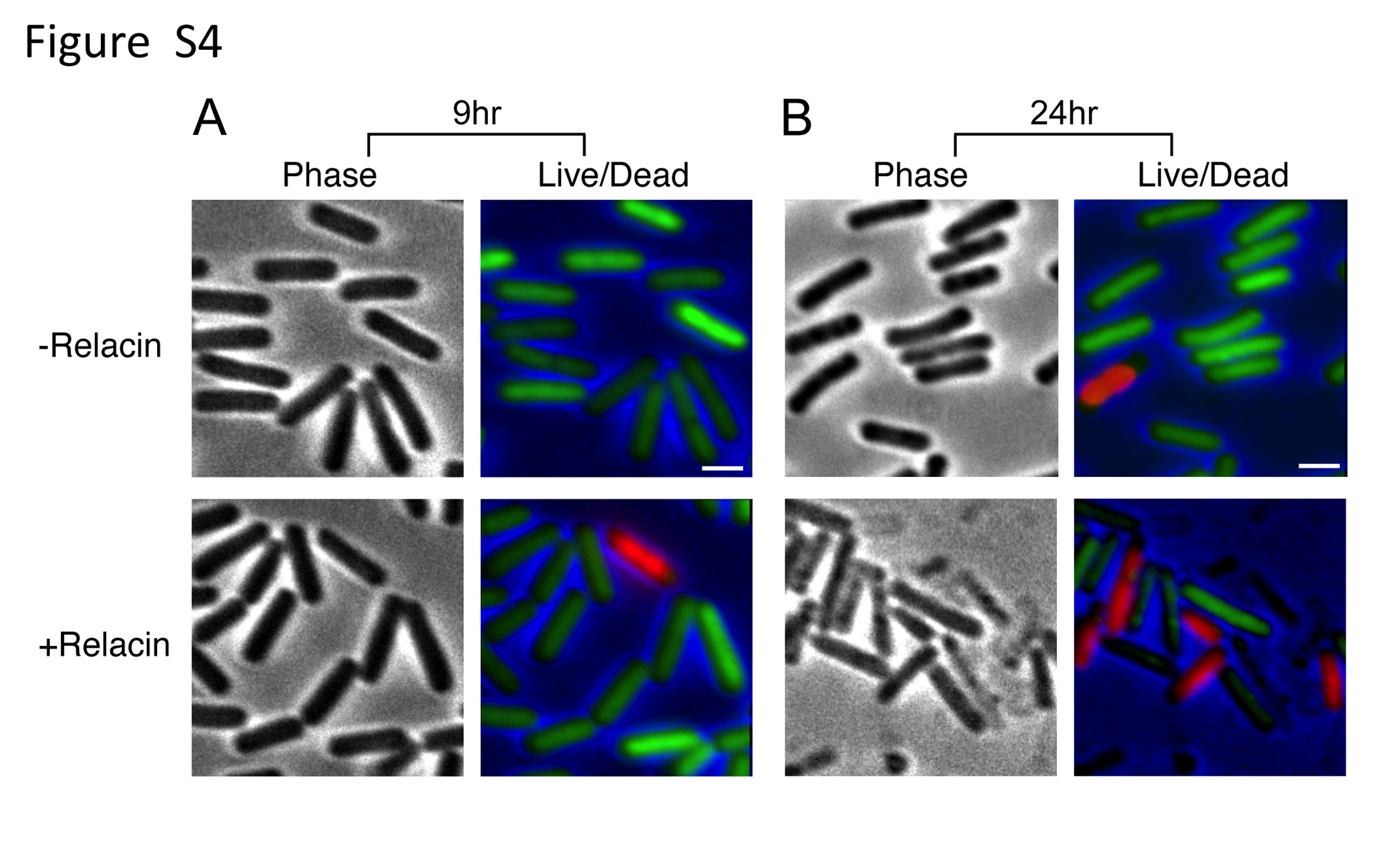

Supplement: Figure S4 — The toxic effect of Relacin is visible. (A–B) B. subtilis (PY79) cells were grown in CH medium at 37°C in the absence (upper panels) and presence (lower panels) of Relacin (1 mM), added at OD600 0.2. Cells were stained with viability indicators SYTO9 (green, highlights live cells) and PI (red, highlights dead cells) at 9 hours (A) and 24 hours (B) of incubation. Shown are phase contrast images (left panels) and Live/Dead overlay fluorescence images (right panels). Of note, some of the disintegrated cells were not stained with any of the dyes. Scale bar corresponds to 1 µm. (TIF) [file ppat.1002925.s004.tif]

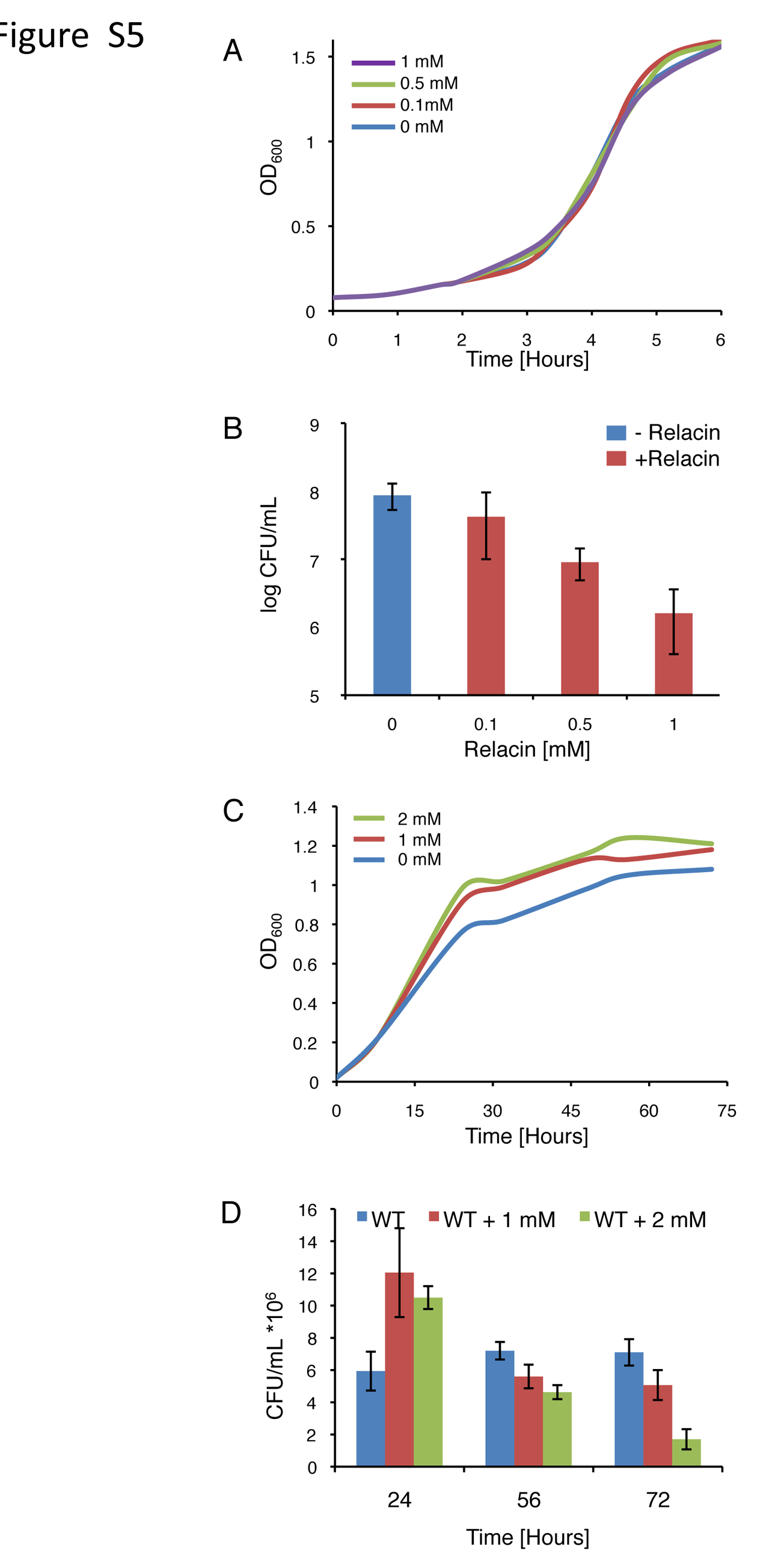

Supplement: Figure S5 — Relacin affects growth and survival of Gram positive bacteria. (A) Effect of Relacin on growth of GAS. Shown are growth curves of wild type GAS (JRS4) cells grown at 37°C without shaking in THY medium in the absence or presence of increasing concentrations of Relacin added at OD600 0.2. (B) Effect of Relacin on survival of GAS. The effect of Relacin, at the indicated concentrations, on survival of GAS (JRS4) cells grown at 37°C without shaking in THY medium was determined by CFU counting of treated and untreated cultures. Relacin was added at OD600 0.2. Shown is a representative experiment, in which SD was calculated from at least three repeats for each concentration. (C) Effect of Relacin on growth of D. radiodurans. Shown are growth curves of wild type D. radiodurans R1 grown in TYG medium at 30°C in the absence or presence of Relacin at the indicated concentrations added at OD600 0.2. (D) Effect of Relacin on D. radiodurans survival. The effect of Relacin, at the indicated concentrations, on survival of wild type D. radiodurans R1 cells grown at 30°C in TYG medium was determined by CFU counting of treated and untreated cultures. Relacin was added at OD600 0.2. Shown is a representative experiment, in which SD was calculated from at least three repeats for each point. (TIF) [file ppat.1002925.s005.tif]

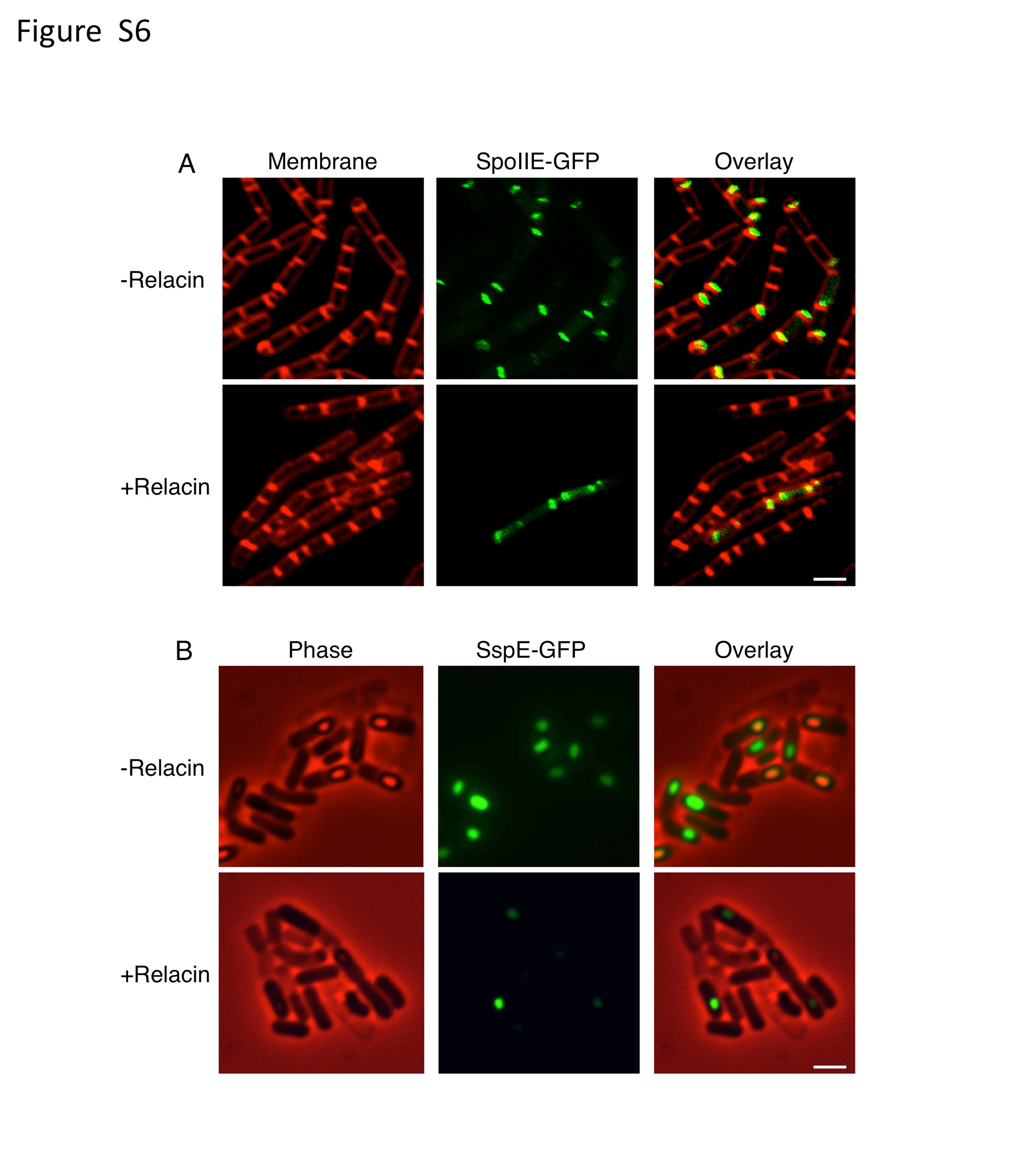

Supplement: Figure S6 — Effect of Relacin on the expression of early and late sporulation-specific proteins. (A) Fluorescence microscopy images of B. subtilis (SB201) cells harboring spoIIE-gfp fusion at t = 2 hr of sporulation, in the absence (upper panels) and presence (lower panels) of Relacin (1 mM), added at time 0 of sporulation. Shown are cells stained with FM4–64 membrane dye (red), SpoIIE-GFP fluorescence (green) and overlay images. Scale bar corresponds to 1 µm. (B) Fluorescence microscopy images of B. subtilis (ES7) cells harboring sspE-gfp fusion at t = 5 hr of sporulation, in the absence (upper panels) and presence (lower panels) of Relacin (1 mM), added at time 0 of sporulation. Shown are phase contrast (red), SspE-GFP fluorescence (green) and overlay images. Scale bar corresponds to 1 µm. (TIF) [file ppat.1002925.s006.tif]
