# Supplementary material for: Relacin, a Novel Antibacterial Agent Targeting the Stringent Response
Source: PLoS Pathog. 2012 Sep 20;8(9):e1002925. doi: 10.1371/journal.ppat.1002925 (PMC3447753; doi:10.1371/journal.ppat.1002925)
Supplement: Text S1 — Supplemental information, including one table as well as additional experimental procedures and references. (DOCX) [file ppat.1002925.s007.docx]

## Supporting Information

**Supporting Information Contains:**

*SI* Materials and Methods

Table S1

*SI* References

***SI* Materials and Methods**

**Synthesis of Relacin**

All chemicals and reagents were purchased from Sigma-Aldrich Ltd. and used without further purification. Ultra-dry solvents stored over molecular sieves under an argon atmosphere were purchased from Acros Ltd. ^1^H and ^13^C NMR data were recorded on a Varian VXR-300 MHz spectrometer equipped with a 5-mm switchable probe. Data were processed using the VNMR software. NMR multiplicities are abbreviated as follows: s (singlet), d (doublet), t (triplet), q (quartet), m (multiplet), br (broad). Electrospray ionization mass spectrometry was carried out using a ThermoQuest Finnigan LCQ-Duo in the positive ion mode. Data were processed using ThermoQuest Finnigan’s Xcalibur^™^ Biomass Calculation and Deconvolution software. High Resolution Mass Spectrometry (HRMS) was carried out with Orbi-trap (Thermo Finnigan) using nanospray attachment. MRFA was used as internal standard. Data were processed using BIOWORKS 3.3 package. The synthetic pathway is depicted in Supplementary Fig. 1.

**Chemical procedures**

**Boc-Gly-OH (1):**

Boc-Gly-OH was prepared as described previously [[1](#_ENREF_1)], yielding colorless crystals: 9.3g, 77% (53 mmol).

^1^H NMR (DMSO-d_6_): δ 5.11 (br s, 1H), 3.76-3.67 (m, 2H), 1.44 (s, 9H)**;** ^13^C NMR (DMSO-d_6_): δ 172.7, 157.4, 79.6, 44.2, 25.3; ESI-MS: Calc: 175.08 Found: 175.98

**Benzyl glycine (2):**

Benzyl Glycine was prepared according to previously reported procedure [[2](#_ENREF_2)] and obtained as a white powder corresponding to the Tosylate salt. 43g, 95% (0.26 mol).

^1^H NMR (DMSO-d_6_): δ 8.11 (s, 3H), 7.69 (d, *J*=7.8 Hz, 2H), 7.31 (m, 5H, (Benzyl)), 6.99 (d, *J*=8.1 Hz, 2H), 4.99 (s, 2H), 3.71 (s, 2H), 2.25 (s, 3H)**;** ^13^C NMR (DMSO-d_6_): δ 168.73, 143.10, 140.51, 135.47, 130.23, 129.70, 129.70, 129.29, 126.23, 69.10, 40.97, 21.35; ESI-MS: Calc: 165.08 Found: 166.01

**Boc-Gly-Gly-OBzl (3):**

Boc-Glycine (5.25 g, 30 mmol) was dissolved in dimethylformamide (800 mL) and coupling reagents HBTU (5.94 g, 45 mmol) and HOBT (17g, 45 mmol) were added. After stirring at room temperature for one hour, compound (**2**) (7.5 g, 45 mmol) and diisopropylethylamine (157 mL, 0.9 mol) were added. After stirring overnight at room temperature the solvents were evaporated and the oily residue was dissolved in ethyl acetate and washed with aqueous citric acid, aqueous sodium bicarbonate and brine. After drying and evaporating the organic solvent, the desired compound was purified by column chromatography (ethyl acetate) and obtained as a white powder. 9.5g, 98% (29.5 mmol).

^1^H NMR (DMSO-d_6_): δ 8.21 (t, *J*=11.3 Hz, 1H), 7.34 (m, 5H), 6.99 (t, *J*=11 Hz, 1H), 5.1 (s, 2H), 3.88 (d, *J*=5.9 Hz, 2H), 3.55 (d, *J*=6.1 Hz, 2H), 1.35 (s, 9H); ^13^C NMR (DMSO-d_6_): δ 176.3, 170.1, 167.4, 157.4, 136.5, 129.1, 128.8, 128.6, 79.6, 66.7, 41.4, 25.3; HRMS: Calc: 323.1601 Found: 323.1602

**H-Gly-Gly-OBzl (4):**

The fully protected dipeptide (**3**) (26.3 g, 0.081 mmol) was dissolved in a solution of trifluoroacetic acid in dichloromethane (200 mL, 50%) and stirred at room temperature for 30 minutes. The solvents were evaporated and ethyl ether was added. The TFA salt precipitates as a white powder. 7.6g, 86% (22.6 mmol)**.**

^1^H NMR (DMSO-d_6_): δ 8.87 (t, *J*=11 Hz, 1H), 8.11 (s, 3H), 7.34 (m, 5H), 5.13 (s, 2H), 4.01 (d, *J*=5.7 Hz, 2H), 3.62 (s, 2H); ^13^C NMR (DMSO-d_6_): δ 176.3, 170.1, 167.4, 136.5, 129.1, 128.8, 128.6, 66.7, 41.4; HRMS: Calc: 223.1077 Found: 223.1077

**2-Isobutyryl-2'-deoxyguanosine (5):**

Compound (**5**) was prepared according to a procedure previously reported [[3](#_ENREF_3)] and obtained as a white powder. 9g, 87% (26.7 mmol).

^1^H NMR (DMSO-d_6_): δ 8.2 (s, 1H), 6.2 (t, *J*=13.5 Hz, 1H), 5.3 (bs, 1H), 4.9 (bs, 1H), 4.3 (m, 1H), 3.8 (q, *J*=11.9 Hz, 1H), 3.5 (m, 2H), 2.7 (m,1H), 2.6 (m, 1H), 2.2 (m, 1H), 1.1 (d, *J*=7 Hz, 6H); ^13^C NMR (DMSO-d_6_): δ 180.04, 154.76, 148.30, 148.00, 137.30, 120.11, 87.66, 82.88, 70.42, 61.41, 39.65, 34.68, 18.77; ESI-MS: Calc: 337.33 Found: 338.21

**Preparation of compound (6):**

Compound (**5**) (200 mg, 0.6 mmol) was suspended in dry acetonitrile (10 mL) and four equivalents of carbonyldiimidazole were added. After stirring overnight at room temperature, the precipitate was filtered and re-suspended in dry dichloromethane (10 mL). Four equivalents of (**4**) and eight equivalents of diisopropylethylamine were added. The suspension was stirred at room temperature for 20 hours. At the end of the reaction, water was added and the organic phase was washed three times with 5% aqueous citric acid (20 mL). After drying and evaporating the organic phase, the crude material was purified by column chromatography (ethyl acetate) obtaining the desired compound as a white powder. 240 mg, 48% (0.28 mmol).

^1^H NMR (DMSO-d_6_): δ 8.36 (m, 2H), 8.26 (s, 1H), 7.7 (t, *J*=12 Hz, 1H), 7.61 (t, *J*=11.8 Hz, 1H), 7.33 (m, 10H), 6.2 (t, *J*=15 Hz, 1H), 5.17 (d, *J*=5.8 Hz, 1H), 5.12 (s, 4H), 4.17 (m, 3H), 3.92 (d, *J*=4.9 Hz, 4H), 3.67 (m, 4H), 2.9 (m, 1H), 2.7 (m,1H), 2.4 (m, 1H), 1.1 (d, *J*=6.9 Hz, 6H)**;** ^13^C NMR (DMSO-d_6_): δ 180.04, 176.3, 170.1, 167.4, 154.76, 148.30, 148.00, 137.30, 136.5, 129.1, 128.8, 128.6, 120.11, 87.66, 82.88, 70.42, 66.7, 61.41, 41.4, 39.65, 34.68, 18.77; HRMS: Calc: 834.3053 Found: 834.3054

**Relacin (7):**

Compound **(6)** (0.5 g, 0.6 mmol) was dissolved in methanol (100 mL). 10% Pd on activated carbon was added cautiously to the solution and the mixture was shaken for three hours under hydrogen atmosphere at 30 psi. The reaction mixture was filtered and evaporated to yield Relacin as off-white foam. 320 mg, 80% (0.49 mmol).

^1^H NMR (DMSO-d_6_): δ 8.26 (s, 1H), 8.17 (m, 2H), 7.69 (t, *J*=12 Hz, 1H), 7.59 (t, *J*=11.8 Hz, 1H), 6.2 (t, *J*=15 Hz, 1H), 5.17 (d, *J*=5.8 Hz, 1H), 4.17 (m, 3H), 3.76 (d, *J*=4.9 Hz, 4H), 3.65 (m, 4H), 2.92 (m, 1H), 2.76 (m,1H), 2.43 (m, 1H), 1.11 (d, *J*=6.9 Hz, 6H)**;** ^13^C NMR (DMSO-d_6_): δ 180.04, 176.3, 170.1, 167.4, 154.76, 148.30, 148.00, 137.30, 120.11, 87.66, 82.88, 70.42, 61.41, 41.4, 39.65, 34.68, 18.77; HRMS: Calc: 654.2114 Found: 654.2114. Elemental analysis calculated for C_24_H_31_N_9_O_13_.2H_2_O: C, 41.80; H, 5.12; N, 18.28. Found: C, 42.63; H, 5.25; N, 18.00.

**Structural model for the interaction of Relacin with Rel/Spo (p)ppGpp synthetase binding pocket**

To investigate the structural basis of the inhibition of Rel/Spo by Relacin, we modeled Relacin into the GDP binding pocket of Rel/Spo from *Streptococcus equisimilis* using computational small molecule docking. The structure of Rel/Spo bound to GDP (pdb id 1vj7) [[4](#_ENREF_4)], was used as template (GDP was removed). Computational small molecule docking was performed using AutoDock Vina [[5](#_ENREF_5)], allowing flexibility to all of the rotatable bonds of the ligand. The docking grid was defined as a 30x30x30 Å grid centered around the position of GDP in the solved crystal structure, with the ‘exhaustiveness’ parameter set to 100. An initial set of 3D conformations of the ligand was created using the MMFF94 force field [[6](#_ENREF_6)], as implemented in the Avogadro molecular viewer (<http://avogadro.openmolecules.net/>). Based on Relacin analogs, we found that the isobutyryl group is critical for Rel/Spo inhibition and therefore chose the best-scoring model, in which the isobutyryl moiety makes significant contacts with the receptor Rel/Spo (ranked second among the models).

**Sporulation conditions and heat resistance assay**

Sporulation of *B. subtilis* cells was induced by resuspension in Sterlini and Mandelstam medium [[7](#_ENREF_7),[8](#_ENREF_8)] of cultures grown in CH medium to an OD_600_ 0.5. After 24 hours, spores were heated at 80ºC for 15 minutes, plated on LB and incubated at 37ºC for 15 hr. Spores of *B. anthracis* were prepared in Difco Sporulation medium [[9](#_ENREF_9)]. After 48 hr, spores were heated at 80ºC for 15 minutes, plated on LB and incubated at 37ºC for 15 hr.

**Biofilm colony and pellicle formation**

For biofilm colony formation on solid medium, cells of *B. subtilis* strain (3610) were grown to exponential phase in LB. 3 μL of culture with or without the addition of 1 mM Relacin were spotted onto solid MSgg medium containing 1.5% Bacto agar, and plates were incubated at 23ºC for 24 hr [[10](#_ENREF_10)]. For pellicle formation in liquid medium, cells were grown to exponential phase in LB and 6 μL of culture were inoculated into 2 mL of MSgg medium with or without the addition of Relacin. Cultures were placed in 12-well plates (Nunc) and incubated at 23ºC for 72 hr.

**Estimating the biofilm biomass by GFP fluorescence measurements**

*B. subtilis* (YA224) cells harboring P*_rrnE_-gfp* fusion were induced to form biofilm in 1 ml of liquid standing culture in the absence or presence of Relacin. After 3 days of incubation, the entire culture (1 ml) was subjected to a treatment of three cycles of FastPrep-24 (MP Biomedicals), setting 6.5 m/s, for 45 s, with 5 min of ice cooling between cycles, in the presence of 0.3 g lysing matrix beads (MP Biomedicals), till the entire culture was homogenized. Next, 200µl of the treated culture were taken to 96-well plates, and GFP fluorescence was measured (excitation: 490 nm; emission: 510 nm) using a Victor3 fluorescence reader (Perkin-Elmer).

**Live/Dead cell viability assay**

Cell viability was visualized using standard Live/Dead staining. Cells were incubated for 5 min in PBS x1 containing a mixture of 10 µg/ml SYTO9 (Invitrogen) and 10 µg/ml PI (Invitrogen) and visualized by fluorescence microscopy.

**Plasmid construction**

**pIK** expressing *his_6_-rel/spo* fusion was constructed by amplifying the *rel/spo* gene from *D. radiodurans* R1 using primers 5'-CGCGGATCCATTGGCTCCCGCTGATGTGAGACAC-3' and 5'-ACGCGTCGACTTATACTAGGCCCGCTTCGCCCCA-3'. The PCR product was digested with *Bam*HI and *Sal*I and cloned in frame into pET28B (His_6_*-*expression vector, Novagen) digested with the same enzymes. pIK was transformed into *E. coli* (BL21 CodonPlus, Stratagene) cells for protein expression.

**Table S1: Bacterial strains**

| **Bacterium** | **Strain**  **Name** | **Genotype** | **Source** |
| --- | --- | --- | --- |
| *B. subtilis* | 3610 | Wild type | [[10](#_ENREF_10)] |
| *B. subtilis* | PY79 | Wild type | [[11](#_ENREF_11)] |
| *B. subtilis* | TW30 | *TrpC2, pheA, sfp^0^, relA::mls* | [[12](#_ENREF_12)] |
| *B. subtilis* | ME215 | *relA::mls* | PY79 was transformed with TW30 genomic DNA |
| *B. subtilis* | PE128 | *amyE::*P*_spoIIQ_-gfp-spc* | [[13](#_ENREF_13)] |
| *B. subtilis* | SB201 | *spoIIE-gfp-kan* | [[14](#_ENREF_14)] |
| *B. subtilis* | ES7 | *sspE-gfp-spc* | Laboratory stock |
| *B. subtilis* | YA224 | *amyE::*P*_rrnE_-gfp-spc* | 3610 was transformed with AR16 genomic DNA [[15](#_ENREF_15)] |
| *B. anthracis* | Sterne 34F2 | pXO1+, pXO2− | A gift from C. Block |
| *E. coli* | W3110 | Wild type | [[16](#_ENREF_16)] |
| *E. coli* | CF9467 | *relA::kan, lacI^q^* | A gift from M. Cashel |
| *D. radiodurans* | R1 | Wild type | [[17](#_ENREF_17)] |
| Group A streptococcus | JRS4 | Wild type | [[18](#_ENREF_18)] |

Except for 3610 and YA224 all *B. subtilis* strains were derivatives of PY79 [[11](#_ENREF_11)]

***SI* References**

1. Brough P, Klumpp C, Bianco A, Campidelli S, Prato M (2006) [60]Fullerene-pyrrolidine-N-oxides. Journal of Organic Chemistry 71: 2014-2020.

2. Sanz-Cervera JF, Blasco R, Piera J, Cynamon M, Ibanez I, et al. (2009) Solution versus fluorous versus solid-phase synthesis of 2,5-disubstituted 1,3-azoles. Preliminary antibacterial activity studies. Journal of Organic Chemistry 74: 8988-8996.

3. Challa H, Bruice TC (2004) Deoxynucleic guanidine: synthesis and incorporation of purine nucleosides into positively charged DNG oligonucleotides. Bioorganic & Medicinal Chemistry 12: 1475-1481.

4. Hogg T, Mechold U, Malke H, Cashel M, Hilgenfeld R (2004) Conformational antagonism between opposing active sites in a bifunctional RelA/SpoT homolog modulates (p)ppGpp metabolism during the stringent response. Cell 117: 57-68.

5. Trott O, Olson AJ (2010) AutoDock Vina: improving the speed and accuracy of docking with a new scoring function, efficient optimization, and multithreading. Journal of Computational Chemistry 31: 455-461.

6. Halgren TA (1996) Merck molecular force field .1. Basis, form, scope, parameterization, and performance of MMFF94. Journal of Computational Chemistry 17: 490-519.

7. Harwood CR, Cutting SM (1990) Modern microbiological methods molecular biological methods for *Bacillus*

8. Sterlini JM, Mandelstam J (1969) Commitment to sporulation in *Bacillus subtilis* and its relationship to development of actinomycin resistance. Biochemical Journal 113: 29-37.

9. Schaeffer P, Millet J, Aubert JP (1965) Catabolic repression of bacterial sporulation. Proceedings of the Natural Academy of Science U S A 54: 704-711.

10. Branda SS, Gonzalez-Pastor JE, Ben-Yehuda S, Losick R, Kolter R (2001) Fruiting body formation by *Bacillus subtilis*. Proceedings of the National Academy of Sciences of the United States of America 98: 11621-11626.

11. Youngman P, Perkins JB, Losick R (1984) Construction of a cloning site near one end of Tn917 into which foreign DNA may be inserted without affecting transposition in *Bacillus subtilis* or expression of the transposon-borne *erm* gene. Plasmid 12: 1-9.

12. Wendrich TM, Marahiel MA (1997) Cloning and characterization of a *relA*/*spoT* homologue from *Bacillus subtilis*. Molecular Microbiology 26: 65-79.

13. Eichenberger P, Fawcett P, Losick R (2001) A three-protein inhibitor of polar septation during sporulation in *Bacillus subtilis*. Molecular Microbiology 42: 1147-1162.

14. Garti-Levi S, Hazan R, Kain J, Fujita M, Ben-Yehuda S (2008) The FtsEX ABC transporter directs cellular differentiation in *Bacillus subtilis*. Molecular Microbiology 69: 1018-1028.

15. Rosenberg A, Sinai, L., Smith, Y., and Ben-Yehuda, S. (2012) Dynamic expression of the translational machinery during *Bacillus subtilis* life cycle at a single cell level. PLoS ONE In Press.

16. Paigen K (1966) Phenomenon of transient repression in *Escherichia coli*. Journal of Bacteriology 91: 1201-1209.

17. White O, Eisen JA, Heidelberg JF, Hickey EK, Peterson JD, et al. (1999) Genome sequence of the radioresistant bacterium *Deinococcus radiodurans* R1. Science 286: 1571-1577.

18. Scott JR, Guenthner PC, Malone LM, Fischetti VA (1986) Conversion of an M- Group-A *Streptococcus* to M+ by transfer of a plasmid containing an M6 gene. Journal of Experimental Medicine 164: 1641-1651.
